# Minimal-Change Disease Secondary to *Borrelia burgdorferi* Infection

**DOI:** 10.1155/2012/294532

**Published:** 2012-02-21

**Authors:** Ewa Kwiatkowska, Edyta Gołembiewska, Kazimierz Ciechanowski, Karolina Kędzierska

**Affiliations:** Department of Nephrology, Transplantology and Internal Medicine, Pomeranian Medical University, 70-111 Szczecin, Poland

## Abstract

Lyme borreliosis is a chronic illness caused by tick-transmitted spirochete *Borrelia burgdorferi*. Borreliosis can be extremely threatening if it is not diagnosed and treated in early stages. Kidneys are not typically involved in the disease. However, in infected dogs, Lyme nephritis is present in 5–10% of cases. It is associated with rapidly progressing renal failure. Histopathological examination shows mesangial proliferative glomerulonephritis with diffuse tubular necrosis, (Dambach et al. (1997)). In available literature, there were reports of human's glomerulonephritis associated with *Borrelia burgdorferi* infection. These cases refer to membranous and mesangial proliferative glomerulonephritis (Kirmizis and Chatzidimitriou (2010), Zachäus (2008), and Kirmizis et al. (2004)). In this paper, we present the case of minimal-change disease (MCD) as a result of *Borrelia burgdorferi* infection.

## 1. Introduction

Lyme borreliosis is a chronic illness caused by tick-transmitted spirochete *Borrelia burgdorferi*. The disease, beginning usually with a typical skin rash, is characterized by a variety of symptoms, including disorders in the musculoskeletal, cardiovascular, and central nervous systems. Tick bites are common, especially in farmers and persons living in endemic areas (Podlaskie Province, Warmińsko-Mazurskie Province, Opolskie Province); however, all Poland can be assumed as endemic area. In recent years, there is increasing trend in borreliosis prevalence. Borreliosis can be extremely threatening if it is not diagnosed and treated in early stages. Classification and staging of Lyme borreliosis according to Asbrink and Hovmark into early borreliosis (localized infection and early disseminated infection) and late borreliosis (chronic infection) is commonly used [1]. *Erythema migrans*, an early manifestation of *Borrelia burgdorferi* dissemination in skin, is the only pathognomonic symptom, present in approximately 50% of patients according to some authors [2], according to others in 70–80% [3].

Kidneys are not typically involved in the disease. However, in infected dogs, Lyme nephritis is present in 5–10% of cases. It is associated with rapidly progressing renal failure. Histopathological examination shows mesangial proliferative glomerulonephritis with diffuse tubular necrosis [4]. In available literature, there were reports of human's glomerulonephritis associated with *Borrelia burgdorferi* infection. These cases refer to membranous and membranoproliferative glomerulonephritis [5–7]. In this paper, we present the case of minimal-change disease (MCD) as a result of *Borrelia burgdorferi* infection.

## 2. Case Report

A 37-year-old patient removed the tick from the skin of the right hip at the end of August 2009. He found the tick after a walk in the forest. Erythema appeared in the place of removed tick in September 2009. At first, erythema was small; however, it quickly grew and had bluish colour. At the end of October (2 months after tick removal) patient went to family doctor and then to infectious disease doctor. On the basis of clinical manifestation and serological tests—the presence of antibodies against *Borrelia burgdorferi* confirmed by Western blot tests—infectious disease doctor diagnosed early limited borreliosis. Since December patient received doxycycline for 30 days in the dose 100 mg twice daily. Since December he observed increasing body weight and abdominal circumference as well as crural oedema. Finally patient was admitted to Internal Medicine ward with massive crural oedema. Erythema migrans on the skin of right hip disappeared soon after the start of doxycycline treatment. Laboratory tests revealed 24-hour proteinuria of 4.5 g, hypercholesterolemia (LDL-cholesterol 10,96 mmol/L), hypoproteinemia (total protein 42 g/L), hypoalbuminemia (17.6 g/L), hypogammaglobulinemia, hyper alpha- and hyper beta-globulinemia. Nephrotic syndrome was diagnosed and its secondary causes were looked for. Autoimmunological, oncological, and infectious causes (other than *Borrelia burgdorferi*) were excluded. Creatinine level was 0.79 mg/dL, creatinine clearance 119 mL/min/1,73 m^2^. C-reactive protein level was 5 mg/L. Angiotensin-converting enzyme inhibitor, statin, and acetylsalicylic acid were added to treatment. In February 2010, patient was admitted to the Department of Nephrology, Transplantology and Internal Medicine, where kidney biopsy was performed. At admission crural oedema, massive proteinuria (12 g/24 hours), hypoproteinemia, and hypoalbuminemia were found. Lipid disorders decreased as a consequence of statin treatment. Kidney biopsy was performed. 14 glomeruli were obtained for examination. In light microscopy no inflammatory infiltration and tubular atrophy were observed. Cross-section of 2 arteries and 2 arterioles revealed no changes. Immunohistochemical examination showed only IgM deposits in interstitium. Kidney biopsy revealed minimal-change disease. Patient was given methylprednisolon intravenously, followed by oral steroids. Currently the course of the disease is observed by both nephrologist and infectious diseases doctor.

## 3. Discussion

Minimal-change diseases remain the cause of 20–25% of primary nephrotic syndrome in adults [8, 9]. It is assumed that defect in the function of T lymphocytes leads to the development of the disease. The mechanism of proteinuria is not known yet; however, it is supposed to be associated with circulating lymphokine which neutralizes negative charge of filtration membrane. This enables protein transfer and leads to podocyte dysfunction [10, 11]. Podocyte damage evolves in each nephrotic syndrome—these highly specialized epithelial cells constitute one of the layers of filtration membrane. Reiser et al. in the series of studies observed that podocytes may acquire functions typical for dendritic cells, B lymphocytes, and other APCs (antigen-presenting cells). They can induce the expression of CD80 protein (B7-1 protein). It is transmembrane protein that provides a costimulatory signal for T-cell activation. The activation of T lymphocyte and its differentiation take place after CD80 binding to CD28 and CTLA-4 receptors on T lymphocyte. Little is known about the expression of CD80 protein on cells other than the ones from bone marrow. In his experiments, Reiser found that lipopolysaccharides administered to mice led to increased expression of CD80 on podocytes and to proteinuria. Such effect was not observed in CD80 knockout mice. The activation of CD80 by polysaccharides can take important part in the pathogenesis of minimal-change disease [12]. Garin et al. found significantly higher urinary excretion of CD80 in patients with MCD relapse compared to subjects with MCD remission, healthy subjects, and patients with other glomerular diseases. Specific ligands binding to TLR (Toll-like receptor) start one of the pathways of CD80 activation. Lipopolysaccharides (LPS) present on the surface of Gram-negative bacteria can play a role of such ligand [13].

Lyme disease is a multisystem disease caused by *Borrelia burgdorferi*. Immunological response plays a key role in the pathogenesis of the disease [14, 15]. Increased expression of costimulating proteins—CD80 and CD86, responsible for T-cell activation and signaling from APC, remains one of the most important factors [16]. Bulut et al. showed that activation of immunological response by spirochete required activation of TLR. *Borrelia burgdorferi* does not have LPS, typical for Gram-negative bacteria, on its surface. However, it does have on its surface different lipoproteins which bind to TLRs, mainly 1, 2, and 4. Surface protein OspA is one of such proteins activating TLRs [17]. The role of these receptors in response to *Borrelia burgdorferi* infection was confirmed by other authors [18, 19].

*Borrelia burgdorferi* can bind to TLRs with lipoproteins present on the surface of the bacteria. This way it activates CD80. In the study by Reiser, he observed the possibility of the activation of costimulatory proteins in podocytes. It seems such activation took place in the presented case. It also seems that similar mechanism leads to transient proteinuria in patients with sepsis caused by Gram-negative bacteria [20]. Surface lipopolysaccharides activate TLR, protein CD80, and in this way activate T lymphocytes which produce lymphokine responsible for proteinuria. It can be confirmed by the fact that in patients with MCD the development of nephrotic syndrome is associated with infection [21].

It is known that not all patients with borreliosis develop nephrotic syndrome. This can be associated with decreased expression of CTLA-4 receptor which binds CD80 and blocks activation of T-cell [22]. It is possible that this phenomenon is associated with other proteins regulating the production of lymphokines.
